# *In Vivo* Evaluation of* Galla chinensis* Solution in the Topical Treatment of Dermatophytosis

**DOI:** 10.1155/2017/3843595

**Published:** 2017-11-12

**Authors:** Kai Sun, Xu Song, RenYong Jia, Zhongqiong Yin, Yuanfeng Zou, Lixia Li, Lizi Yin, Changliang He, Xiaoxia Liang, Gang Ye, Guizhou Yue, Xinghong Zhao, Jiankang Yu

**Affiliations:** ^1^Natural Medicine Research Center, College of Veterinary Medicine, Sichuan Agricultural University, Chengdu 611130, China; ^2^Key Laboratory of Animal Disease and Human Health of Sichuan Province, Sichuan Agricultural University, Chengdu 611130, China; ^3^College of Science, Sichuan Agricultural University, Ya'an 625014, China

## Abstract

**Aim:**

Dermatophytosis is one of the main fungal diseases in humans and animals all over the world.* Galla chinensis*, a traditional medicine, has various pharmacological effects. The goal of this study was to evaluate the treatment effect of* Galla chinensis* solution (GCS) on dermatophytosis-infected dogs (*Microsporum canis*,* Microsporum gypseum*, and* Trichophyton mentagrophytes*, resp.).

**Methods:**

The treatment effects of GCS were evaluated by mycological cure rates and clinical score comprised of three indices, including inflammation, hair loss, and lesion scale.

**Results:**

The results showed that, in the three models of dermatophytosis, GCS significantly (*P* < 0.05) improved skin lesions and fungal eradication. GCS (10% and 5%) had higher efficacy compared to the positive control (Tujingpi Tincture). The fungal eradication efficacy exceeds 85% after treatment with GCS (10%, 5%, and 2.5%) on day 14.

**Conclusion:**

The GCS has antidermatophytosis effect in dogs, which may be a candidate drug for the treatment of dermatophytosis.

## 1. Introduction

Dermatophytosis is one of the main fungal diseases of humans and animals all over the world, and the major cause is associated with fungal infection, such as genera* Microsporum, Trichophyton*, and* Epidermophyton* [1, 2]. This disease is characterized mainly by invading keratinized tissues, such as hair, skin, and nails, and producing multifocal alopecia, scaling, and circular lesions. It is often painful and has a negative influence on the appearance of animals [3]. It was reported that the rates of infection (*M. canis*,* M. gypseum*, and* T. mentagrophytes*) were 77%, 10%, and 7% in animals (dogs and cats), respectively [4]. At present, there are many chemically synthesized antidermatophytic drugs, including polyenes, fluoropyrimidine, azole, allylamines, and triazole [5]. However, there is some potentially worrying trend in relapses, drug resistance, and severe side effects, including gastrointestinal complications and high toxicity [5, 6]. Therefore, it is urgent to develop new drugs for treatment of dermatophytosis.

Traditional Chinese medicines (TCM) possess widely pharmacological activities [7, 8].* Galla chinensis* is a traditional Chinese herbal medicine and mainly grown in Sichuan Province, China. It is formed from the* sumac aphid Baker* (mainly* Melaphis chinensis* bell) parasitizing the plants of* Rhus chinensis*,* Rhus potaninii *Maxim., and* Rhus punjabensis* [9].* Galla chinensis* is usually harvested in autumn, and, before medicinal use, it needs to be dried to remove the larvae. The main ingredient of* Galla chinensis* is tannin acid, which can exceed 70% of its weight [10].* In vitro* and* in vivo* studies have demonstrated its pharmacological effects, including spermicidal, antibacterial, antifungal, and antioxidant activities [11]. In China,* Galla chinensis* is widely used as antifungal drugs in aquaculture [12]. However, treatment effect of* Galla chinensis* against dermatophytosis in human and animals has not yet been reported. Hence, we developed a* Galla chinensis* solution (GCS), and the activity of GCS against* M. canis, M. gypseum*, and* T. mentagrophytes* was evaluated on dogs, respectively.

## 2. Materials and Methods

### 2.1. Plant Materials

*Galla chinensis* (number 160512) was bought from the Fangsheng Biotechnology Co., Ltd. (Gallnut; Baoji, China), and identified by Dr. Lixia Li (Sichuan Agricultural University, Chengdu, China).* Galla chinensis* was extracted by boiling with distilled water for 2 h (1/15 (w/v)). The extracts were filtered and concentrated. Finally, the tannin extract powder was obtained by lyophilization [13–15].

### 2.2. Drugs

The* Galla chinensis* solution (2.5%, 5%, and 10%) was prepared in Natural Medicine Research Center, Sichuan Agricultural University (Chengdu, China), by dissolving* Galla chinensis* and appropriate ingredients in distilled water, and ingredients include stabilizer (35% glycerinum), antioxidants (0.1% sodium metabisulfite), and transdermal agent (5% propylene glycol and 3% azone). The positive control drug, Tujingpi Tincture (number 151225), was bought from the Guangzhou Baiyun Mountain Jingxiutang Pharmaceutical Co., Ltd. (Guangzhou, China).

### 2.3. Fungal Organism

*M. canis* (ATCC 8137),* M. gypseum* (ATCC 14683), and* T. mentagrophytes* (ATCC 32457) were bought from the inquiry network for microbial strains of China (Beijing, China). The molecular and morphology identification (DNA sequences, the spore morphology, and colony characters) were confirmed by Dr. Xiaoxia Liang (Sichuan Agricultural University, Chengdu, China).

### 2.4. Animals

150 dogs (*Canis lupus familiaris*, average weight 6 ± 2 kg) without dermatophytosis were maintained in the animal houses of College of Veterinary Medicine (Sichuan Agricultural University; Ya'an, China). The experimental protocol was approved by the National Institute of Ethics Committee at Sichuan Agricultural University [approval number SYXK (Sichuan) 2014-187]. The animals were maintained at 12 h light/dark cycle and they were fed rationed commercial dog food and allowed access to sterilized water. The animals were allowed to acclimate for 7 days.

### 2.5. Optimization of Inoculum Concentration

Animal infection was performed following the previous methods [16, 17]. The three fungi were grown on Sabouraud dextrose agar (SDA) medium plates at 28°C for 14–21 days (*M. canis* and* M. gypseum* for 21 days and* T. mentagrophytes* for 14 days, resp.). The conidia of fungus were collected by scraping the SDA surface with a sterilized loop from the cultured material into normal saline, and then vibrated by vortex mixer and diluted with RPMI 1640 medium. The number of conidia in suspensions was measured and adjusted to 1 × 10^7^ CFU/mL, 1 × 10^6^ CFU/mL, 1 × 10^5^ CFU/mL, and 1 × 10^4^ CFU/mL for* M. canis* and* T. mentagrophytes*, respectively, and to 1 × 10^6^ CFU/mL, 5 × 10^5^ CFU/mL, 1 × 10^5^ CFU/mL, and 5 × 10^4^ CFU/mL for* M. gypseum*, respectively [3, 12, 18]. Dogs were randomized into twelve groups (one dog in each group). The skin of these dogs on both sides of the back was clipped by an electric shaver (2.5 cm × 2.5 cm) under anesthesia, respectively. The 0.1 mL conidia solution was smeared on this area. After inoculation for 10 days, the degree of infection was observed. It is showed that more severe infection appeared with the increased concentration of the inoculum. According to the principle that the minimum concentration which can cause clear lesions was chosen for experiments, the concentrations of the fungi were determined as 1 × 10^6^ CFU/mL for* M. canis*, 5 × 10^5^ CFU/mL for* M. gypseum*, and 1 × 10^6^ CFU/mL for* T. mentagrophytes*.

### 2.6. Treatment

On day 10 postinoculation, the experimental animals without any accidental damage to skin were selected. Each model included five groups (ten animals for each group): (i) untreated group, (ii) 10% GCS group, (iii) 5% GCS group, (iv) 2.5% GCS group, and (v) positive control (Tujingpi Tincture). The drugs were applied topically to the infected area (1 mL) once a day for 14 days.

### 2.7. Clinical Evaluation

Clinical assessment was performed though scoring of lesions on 0th, 7th, and 14th days posttreatment according to the method previously described [16, 19, 20]. The main criteria for the evaluation contained three points: inflammation, scale, and hair loss. The inflammation was scored from 0 to 3 as follows: 0: no signs of infection; 1: few slightly erythematous places on the skin; 2: erythematous with papulovesicular areas; 3: large areas of marked erosion and incrustation in places. The infection scale was scored from 0 to 3 as follows: 0: no scale of infection; 1: few slightly scale areas on the skin; 2: medium degree of scale areas; 3: large areas of scale. The hair loss was scored from 0 to 3 as indicated: 0: no loss of hair; 1: loss of hair < 20%; 2: 20% < loss of hair < 40%; 3: loss of hair > 40%. The total score of each animal was obtained by adding the scores of inflammation, scale, and hair loss together, and the maximum score per animal was 18.

### 2.8. Mycological Evaluation

To evaluate the mycological cure rate of GCS, the infected area (2.5 cm × 2.5 cm) on both sides of the back of each dog was divided into four equal quadrants (8 samples per animal). Hair and skin scrap samples from each quadrant of each animal were plucked using a sterile lancet under a light microscope after distributing in 10% KOH. The microscopic result is generally considered positive when finding arthroconidia, conidia, or hyphae. The samples were incubated at 30°C for 7 days. Species identification was done on the basis of the morphology of colonies as previously described [21, 22]. A negative result of microscopy and culture were considered mycological cure. The effectiveness of GCS was assessed with scores ranging from 0 to 9 based on the number of fungal burden of skin samples of each animal. Percent efficacy rates were calculated as follows:

(1) $$\begin{array}{c} \text{Percent efficacy rates}=100-T\times \frac{100}{C}, \end{array}$$

where *T* is the number of fungal burdens of treated group; *C* is the number of fungal burdens of untreated group.

### 2.9. Statistical Analyses

All results were calculated as the means and standard (M ± SD). The results were analyzed for statistical significance by one-way analysis of variance (ANOVA) followed by Student's *t*-test; *P* < 0.05 was considered statistically significant.

## 3. Results

### 3.1. Clinical Evaluation

**Figure 1:**
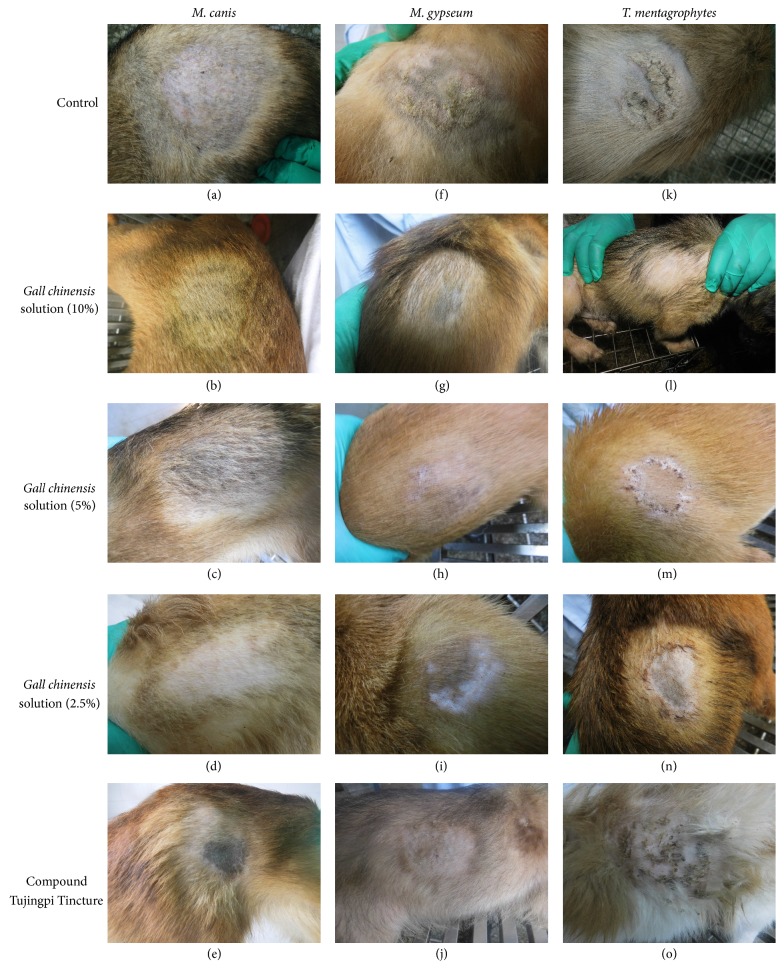
Clinical observation of* Galla chinensis* solution and Tujingpi Tincture against dermatophytosis caused by* M. canis* (a–e),* M. gypseum* (f–j), and* T. mentagrophytes* (k–o) in dogs. (a, f, and k) control. (b, g, and l) 10% GCS group; (d, i, and n) 2.5% GCS group.

**Figure 2:**
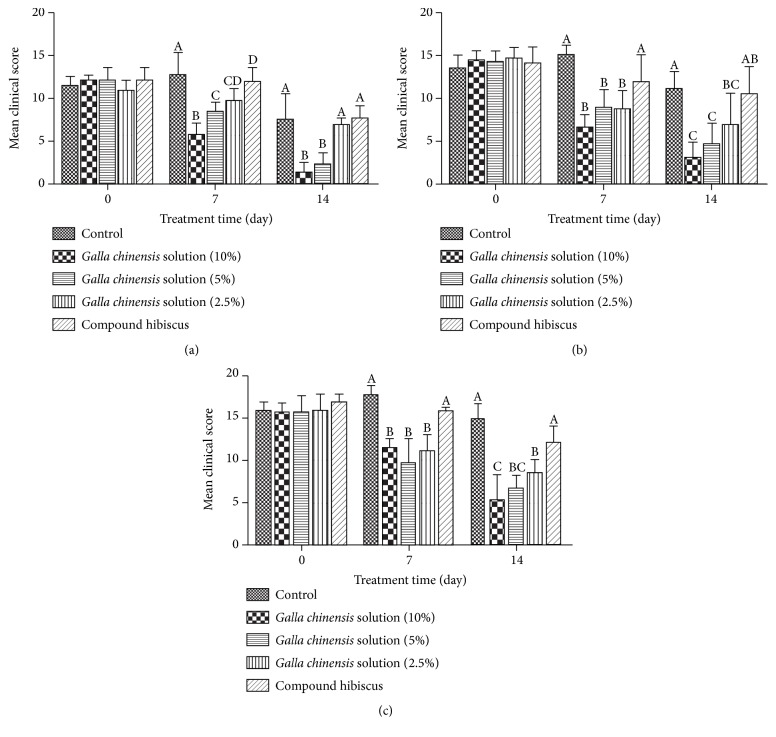
Clinical scores of skin lesions in dogs infected with* M. canis* (a),* M. gypseum* (b), and* T. mentagrophytes* (c). Values represented are the mean ± SD (*n* = 10). Significant differences are mentioned with different alphabets at the level of *P* < 0.05.

All animals were monitored for clinical signs thrice a day throughout the study. High clinical scores indicated severe skin lesions caused by fungal infection. Marked lesions, such as hair loss, scaly skin, and visible papulovesicle of the affected area, appeared on day 10 postinfection and reached highest degree on day 14. Although the damage of control group has been reduced after treatment for 7 days, the lesion was still obvious through 14-day treatment. The results (Figures 1 and 2) showed that GCS could improve skin lesions in a dose-dependent manner in the fungi-infected dogs.

In* M. canis*-induced dermatophytosis test, significant (*P* < 0.05) reduction of the average lesion score treated with GCS (10% and 5%) was observed when compared with control group and Tujingpi-treated group (Figure 2(a)), and all the animals had a complete remission of clinical signs after treatment for 14 days (Figures 1(b) and 1(c)).

In* M. gypseum*-infected and* T. mentagrophytes*-infected dermatophytosis, the GCS (10%, 5%, and 2.5%) significantly (*P* < 0.05) reduced clinical scores compared with the control group (Figure 2). The high dose of GCS (10%) had a complete remission of clinical signs. Most of the skin lesions have been restored, but the hair loss still did not recover completely in the medium (5%) and low (2.5%) doses of GCS (Figures 1(h), 1(i), 1(m), and 1(n)). The positive drug (Tujingpi Tincture) did not significantly (*P* > 0.5) reduce clinical scores, which may be due to the strong irritation of the drug, and led to the increase of scaly skin.

### 3.2. Mycological Evaluation

The mycological cure rate was assessed after treatment for 7 days and 14 days, respectively (Table 1).

In* M. canis*-infected test, compared with the untreated control, the mycological efficacy rates of GCS-treated (10%, 5%, and 2.5%) groups and Tujingpi Tincture-treated group were 87.50%, 75.0%, 72.5%, and 70.0%, respectively, after treatment for 7 days. Those treated with GCS (10%, 5%, and 2.5%) possessed higher mycological cure efficacy (97.5%, 92.5%, and 90.0%, resp.), which is higher than Tujingpi Tincture (87.5%) on day 14 posttreatment.

In* M. gypseum*-infected test, compared with the control group, the mycological efficacy of GCS (10%, 5%, and 2.5%) was greatly improved (80%, 70%, and 60%, resp.) after treatment for 7 days and the Tincture was 70%. Treatment with GCS (10%, 5%, and 2.5%) resulted in a high mycological cure (95.0%, 92.5%, and 90.0%, resp.) after 14-day treatment. In comparison, the efficacy rate of Tujingpi Tincture was 87.5%.

In* T. mentagrophytes-*infected test, GCS and Tujingpi Tincture could significantly eliminate the fungus. The efficacy rates of GCS (10%, 5%, and 2.5%) were 80%, 70%, and 60% on day 7 posttreatment, respectively. All GCS formulations had mycological efficacy greater than 90% on day 14 posttreatment compared to the control group. The efficacy rates of Tujingpi Tincture were 60% and 85% on days 7 and 14 posttreatment, respectively.

## 4. Discussion

Consideration of damage of dermatophytosis to humans and animals is essential to develop more safe and effective new drugs. There were many reports regarding TCM that exhibited antifungal activity in vitro, such as forsythia, polygonum multiflorum, and matrine [23]. However, few studies were conducted on animal model. The dog dermatophytosis model used in this study is highly reproducible and is frequently used to study antidermatophytosis pathogenesis [24]. Dermatophytosis is mainly caused by* Trichophyton rubrum*,* M. canis*,* M. gypseum*, and* T. mentagrophytes*, which usually invades keratinized structure and infects the skin, hair, and nails of animals [2]. Although* T. rubrum* is one common species of dermatophytes, the susceptibility of* T*.* rubrum* to* Galla chinensis *is higher than that of* T. mentagrophytes* [25, 26]. Therefore, GCS would be expected to be more potent in clinical use than the potency exhibited in the present study.

The antifungal activity of* Galla chinensis* in vitro has been documented in previous studies, which showed that the minimal inhibitory concentration (MIC) of* Galla chinensis* for* M. canis*,* M. gypseum*, and* T. mentagrophytes* was 6.25 mg/mL, 12.5 mg/mL, and 6.25 mg/mL, respectively [27, 28]. In this study, antifungal activity of GCS was evaluated in dogs for confirming whether* GCS* could be used to treat dermatophytosis. Our results indicated that GCS had potent antidermatophytosis effects in dogs, which suggested that GCS exhibited high possibility in clinical use. The positive control drug used in this study was Tujingpi Tincture, which is approved by China Food and Drug Administration in China. Tujingpi Tincture made by the extract from* pseudolaricis cortex* in combination with benzyl acid and salicylic acid. It possessed potent antifungal activity and is widely used in the treatment of dermatophytosis [29, 30]. The results of this study showed that GCS had higher activity against dermatophytosis on dogs than Tujingpi Tincture.

In the untreated group, the average scores of skin lesions reached maximum on day 17 postinfection. Then, the average scores were slightly decreased on day 21 postinfection. This finding might be attributed to the immune response of animals to* M. canis, M. gypseum*, and* T. mentagrophytes* which led to self-healing. However, serious lesions observed on day 21 postinfection suggested that dermatophytosis belongs to chronic diseases. With treatment with GCS, the lesions were decreased markedly on day 7, and the animals were almost recovered from dermatophytosis on day 14.

In this study, mycological cure rate is used to reflect elimination of fungi in dogs. The results showed that GCS had a significant eliminating efficacy, which improved with the extension of treatment time. Almost complete eradication of fungi by GCS was detected after a 14-day treatment (more than 90%). The high (10%) and medium (5%) doses of GCS had higher activity than Tujingpi Tincture. The efficacy of GCS at 2.5% was equal to that of Tujingpi Tincture. The result indicated that GCS was a promising drug candidate to treat dermatophytosis. The antidermatophytosis of GCS was not only due to the ability of inhibiting fungal growth in vitro, but also due to the increased permeability of skin to transdermally administered active agents which is caused by propylene glycol and azone in GCS [26, 31, 32].

In conclusion,* Galla chinensis* solution is a potent antifungal agent* in vivo* for dermatophytosis. It could be used for treatment of topically fungal infections.

## Figures and Tables

**Table 1:** Efficacy rates of *Galla chinensis* solution and Tujingpi Tincture on clearance of the fungi in dogs.

Species and group number	Mycological efficacy (%)	
	7 days	14 days
*M. canis*		
10% *Galla chinensis* solution	87.5	97.5
5% *Galla chinensis* solution	75.0	92.5
2.5% *Galla chinensis* solution	72.5	90.0
Compound Tujingpi Tincture	70.0	87.5
*M. gypseum*		
10% *Galla chinensis* solution	80.0	95.0
5% *Galla chinensis* solution	70.0	92.5
2.5% *Galla chinensis* solution	60.0	90.0
Compound Tujingpi Tincture	60.0	87.5
*T. mentagrophytes*		
10% *Galla chinensis* solution	82.5	92.5
5% *Galla chinensis* solution	67.5	90.0
2.5% *Galla chinensis* solution	62.5	85.0
Compound Tujingpi Tincture	60.0	85.0
